# Supplementary material for: The Automatic Solution of Macromolecular Crystal Structures via Molecular Replacement Techniques: REMO22 and Its Pipeline
Source: Int J Mol Sci. 2023 Mar 23;24(7):6070. doi: 10.3390/ijms24076070 (PMC10094557; doi:10.3390/ijms24076070)
Supplement: Supplementary file 1 [file ijms-24-06070-s001.zip › ijms-2203648-supplementary.pdf]

International Journal of *Molecular Sciences*

SUPPLEMENTARY MATERIALS

Article

The Automatic Solution of Macromolecular Crystal Structures via Molecular Replacement Techniques: REMO22 and Its Pipeline

Table S1. The PDB codes of the target structures (Tar.) and of the corresponding MR models (Mod.) used in our experimental tests. Dashes indicate models designed by the original authors, which are available on request.

	**Tar.**	**Mod.**	**Tar.**	**Mod.**	**Tar.**	**Mod.**	**Tar.**	**Mod.**	**Tar.**	**Mod.**	**Tar.**	**Mod.**
**PH**	1a6m	1mbc	1aki	2ihl	1bxo	1er8	1bxo	1er8	1kf4	7rsa	1kf4	7rsa
	1lat	1glu	1lys	2ihl	1na7	1m2r	1na7	1m2r	1tgx	1cdt	1tp3	1be9
	1xyg	1vkn	1ycn	1n00	1yxa	1qlp	1zs0	1i76	2a03	1isc	2a46	1g7k
	2a4k	1uls	2ah8	1ema	2ayv	1x23	2b5o	1b2r	2f53	2bnr	2f84	2aqw
	2fc3	1xbi	2gq3	1n8i	2h8q	1g7k	2hyw	1xjl	2i3p	1g9y	2iff	1hem
	2o3k	1ysb	2oka	2obk	2omt	1o6s	2otb	1zux	2p0g	2oka	2pby	1mki
	2qu5	2p2i	2sar	---	6ebx	3ebx	6rhn	4rhn				
**PD**	3nng	1k3i	3npg	3l5o	3nr6	---	3o8s	2b0v	3on5	2we8	3q6o	3llk
	3tx8	1vgy	3zyt	---	4e2t	2qlm	4fqd	2yvw	1cgn	2ccy	1cgo	2ccy
	1e8a	1mho	2f8m	1uj5	5ww0	3kma						
**PG**	1vkf	3kts	1vki	1vjf	1vl2	1kh1	1vl7	3tgv	1vlc	3u1h	2wu6	1z57
	2x7h	2wek	3e49	3e02	3gp0	1cm8	3h9e	2vyv	3h9r	1b6c	3khu	2q3e
	3l23	3obe	3llx	3wqc	3m7a	3pjy	3mbj	3h74	3mcq	5cm7	3mdo	3kiz
	3mz2	2pz0	3nyy	4qp5	3obi	3nrb	3oz2	4opc	3p94	4iyj	3ufi	4k4k
	3us5	2pe8	4e2e	3iqu	4ef2	4got	4ezg	4l3f	4fvs	3fzx	4gbs	3u22
	4gcm	5uwy	4ler	3ejn	4mru	4f53	4ogz	1uas	4ouq	4orl	4q1v	2z3z
	4q34	1qlw	4q34	1qlw	4q6k	4fj6	4q9a	5bn1	4qjr	1yow	4qni	1yok
	4r0k	3npf	4rvo	2y4o	4rwv	1ymt	4yod	3eur				
**DNA**	1s45	244d	1s45	244d	2b1d	423d	2htt	1xam	3ce5	1k8p	3eil	463d
	3gom	1nvy	3goo	1nvy	3n4o	1dnh	3tok	2org	4gsg	2org	4l24	1bna
	4ltl	3qrn	4ms5	3qrn	4wo3	3qxr	4xqz	5ihd	4zym	4rip	5cv2	4rip
	5i4s	476d	5ihd	2dcg	5j0e	463d	5ju4	1d29	5lj4	463d	5mvt	5mvl
	5nt5	5ju4	5t4w	5ju4	5tgp	1dns	5ua3	1xav_2	6f3c	3np6	6h5r	3r6r
	6tzq	6tzr										
**RNA**	1iha	165d	1lc4	1j7t	1mwl	1lc4	1q96	483d	1z7f	1yrm	2a0p	259d
	2fd0	2fcy	2pn4	2pn3	3d2v	2cky	3fs0	3ftm	3owi	3owz	3oxm	3owi
	3s49	246d	3td1	1mwl	4enc	4enb	4jab	246d	5fj0	4cs1	5kvj	3nd3
	5l4o	3cw5	5nz6	5nwq	5ux3	4fnj	5uz6	5ux3	5zeg	1j7t	6az4	4fnj
	6cab	5dhc										

Table S2. The crystallographic residuals R and average phase error $<|\Delta\phi|>$ obtained by running the three MR test programs in default conditions. $<|\Delta\phi|>_R$ and R_R refer to REMO22, $<|\Delta\phi|>_P$ and R_P to PHASER, $<|\Delta\phi|>_M$ and R_M to MOLREP. The data for the first structure of each subset (PH, PD, PG, DNA, RNA in the order) are in bold. Dashes correspond to cases in which PHASER stops prematurely while trying to estimate the number of chains in the target asymmetric unit.

PDB	$<\|\Delta\phi\|>_R$	R_R	$<\|\Delta\phi\|>_P$	R_P	$<\|\Delta\phi\|>_M$	R_M	PDB	$<\|\Delta\phi\|>_R$	R_R	$<\|\Delta\phi\|>_P$	R_P	$<\|\Delta\phi\|>_M$	R_M
**1a6m**	**26**	**29**	**27**	**30**	**26**	**30**	1aki	22	22	22	23	22	23
1bxo	25	31	34	35	31	35	1dy5	15	22	16	22	90	43
1kf4	12	19	13	19	11	20	1kqw	33	31	35	33	40	34
1lat	58	45	73	54	66	49	1lys	29	22	29	23	59	42
1na7	32	24	35	27	35	27	1s31	34	28	38	31	38	31
1tgx	48	43	73	52	40	37	1tp3	24	28	25	28	25	28
1xyg	43	32	49	36	53	39	1ycn	32	25	62	46	35	28
1yxa	44	35	79	52	74	50	1zs0	31	29	31	30	31	30
2a03	33	30	37	34	37	32	2a46	40	37	50	42	49	42
2a4k	39	31	67	46	45	35	2ah8	33	28	58	45	33	29
2ayv	34	27	41	32	37	29	2b5o	39	28	62	46	46	37
2f53	30	24	34	26	34	26	2f84	37	31	40	32	40	32
2fc3	40	34	44	37	44	37	2gq3	33	25	54	40	33	26
2h8q	35	28	59	47	36	30	2hyw	89	50	31	25	31	25
2i3p	31	26	31	27	30	24	2iff	75	39	67	45	68	45
2o3k	26	23	27	25	28	25	2oka	28	23	27	24	27	24
2omt	31	25	32	25	32	26	2otb	37	31	38	32	42	33
2p0g	37	32	90	55	59	42	2pby	45	35	82	51	77	49
2qu5	27	22	26	23	26	24	2sar	43	39	67	50	49	41
6ebx	24	23	55	45	27	28	6rhn	27	25	28	26	28	27
**3nng**	**71**	**43**	**77**	**52**	**89**	**53**	3npg	80	45	84	50	89	50
3nr6	88	54	89	56	87	57	3o8s	59	45	90	54	89	53
3on5	51	39	69	47	70	48	3q6o	73	45	80	51	83	55
3tx8	69	42	78	51	85	52	3zyt	80	49	86	55	85	55
4e2t	29	28	58	43	53	38	4fqd	78	43	79	49	81	50
1cgn	55	40	74	49	80	52	1cgo	71	47	79	51	76	50
1e8a	39	33	73	48	54	40	2f8m	42	36	68	51	51	41
5ww0	56	42	72	50	75	51	**1vkf**	**90**	**53**	**84**	**53**	**80**	**51**
1vki	39	36	82	53	82	52	1vl2	36	33	66	49	63	45
1vl7	39	33	72	49	47	38	1vlc	32	31	44	36	52	40
2wu6	34	30	40	32	44	35	2x7h	39	24	54	40	34	24
3e49	44	36	71	49	38	30	3gp0	44	35	60	44	68	47
3h9e	28	25	56	45	28	26	3h9r	50	36	54	39	56	40
3khu	31	26	53	38	57	40	3l23	41	34	51	38	56	39
3llx	37	36	43	38	45	38	3m7a	41	35	73	49	53	40
3mbj	47	36	67	44	67	44	3mcq	47	40	73	50	74	52
3mdo	31	28	60	45	32	29	3mz2	89	48	89	51	89	51
3nyy	65	48	74	51	80	51	3obi	89	49	78	50	71	47
3oz2	43	41	74	52	81	52	3p94	48	37	72	49	45	34
3ufi	53	42	79	51	75	51	3us5	38	39	47	43	49	43
4e2e	32	28	36	30	35	31	4ef2	36	30	65	48	53	39

4ezg	25	26	26	27	57	40	4fvs	89	53	80	54	89	52
4gbs	37	32	64	49	41	36	4gcm	33	30	68	49	38	33
4ler	30	31	35	32	37	32	4mru	49	34	72	47	53	36
4ogz	46	36	73	47	58	41	4ouq	25	28	28	29	29	30
4q1v	46	35	54	39	56	39	4q34	42	40	72	52	75	53
4q53	35	38	58	48	42	40	4q6k	32	27	71	48	36	28
4q9a	90	49	71	51	61	44	4qjr	32	28	37	31	36	31
4qni	45	35	75	51	69	48	4r0k	28	25	28	25	47	37
4rvo	89	46	81	52	71	48	4rwv	30	29	37	32	37	33
4yod	63	45	66	46	68	47	**1s45**	**65**	**42**	**69**	**49**	**64**	**45**
1s47	65	43	68	51	88	51	2b1d	79	46	84	53	87	55
2htt	41	29	---	---	44	34	3ce5	47	39	50	40	63	46
3eil	41	31	65	56	84	55	3gom	40	31	59	47	86	55
3goo	35	28	44	38	37	31	3n4o	27	22	27	23	30	25
3tok	46	35	53	40	51	39	4gsg	49	34	---	---	57	36
4l24	38	21	50	43	37	24	4ltl	57	44	57	48	62	48
4ms5	69	49	87	57	86	55	4wo3	66	41	70	47	86	48
4xqz	44	32	46	33	88	45	4zym	88	51	74	54	87	56
5cv2	90	50	62	53	89	54	5i4s	34	25	---	---	55	35
5ihd	40	34	45	36	88	52	5j0e	49	35	76	53	75	50
5ju4	25	27	28	28	89	57	5lj4	28	26	---	---	46	34
5mvt	28	29	46	38	55	41	5nt5	21	26	25	29	21	28
5t4w	21	25	49	42	46	36	5tgp	22	27	57	46	89	53
5ua3	60	43	80	50	86	53	6f3c	51	42	51	46	70	52
6h5r	55	40	67	50	79	53	6tzq	38	36	34	37	40	38
**1iha**	**40**	**34**	**41**	**35**	**87**	**51**	1lc4	29	24	43	39	35	28
1mwl	29	25	46	41	35	29	1q96	89	52	89	57	89	55
1z7f	32	32	34	33	38	34	2a0p	28	27	30	28	30	29
2fd0	33	32	36	33	55	39	2pn4	35	33	61	51	45	40
3d2v	31	27	65	49	57	41	3fs0	30	23	---	---	55	37
3owi	31	28	50	45	39	35	3oxm	21	22	22	30	20	23
3s49	28	31	90	57	89	53	3td1	37	32	66	51	80	52
4enc	28	25	30	31	30	26	4jab	40	27	50	37	44	33
5fj0	90	51	72	56	80	57	5kvj	52	30	72	49	55	28
5l4o	41	32	48	40	64	46	5nz6	27	23	40	37	28	27
5ux3	90	51	89	58	89	58	5uz6	33	34	77	51	89	52
5zeg	74	48	88	56	89	55	6az4	42	36	59	49	44	38
6cab	59	45	58	50	61	50							

Table S3. The MA values obtained by the pipelines REMO22+SYNERGY+CAB (MA_{RSC}), PHASER+SYNERGY+CAB (MA_{PSC}) and MOLREP + SYNERGY + CAB (MA_{MSC}), for each test structure. Data for the first structure of each subset (PH, PD, PG, DNA, RNA in the order) are in bold. Dashes indicate cases where PHASER stops prematurely while trying to estimate the number of chains in the target asymmetric unit.

PDB	MA_{RSC}	MA_{PSC}	MA_{MSC}	PDB	MA_{RSC}	MA_{PSC}	MA_{MSC}	PDB	MA_{RSC}	MA_{PSC}	MA_{MSC}
**1a6m**	**84**	**84**	**84**	1aki	92	93	93	1bxo	91	90	91
1dy5	85	85	4	1kf4	86	87	86	1kqw	91	94	94
1lat	23	7	25	1lys	87	85	83	1na7	80	81	83
1s31	77	79	78	1tgx	87	6	84	1tp3	91	89	87
1xyg	89	90	89	1ycn	82	30	81	1yxa	91	7	89
1zs0	89	87	88	2a03	94	94	92	2a46	92	92	92
2a4k	88	81	88	2ah8	93	92	94	2ayv	90	89	89
2b5o	75	33	77	2f53	87	87	86	2f84	87	89	85
2fc3	92	92	91	2gq3	87	85	88	2h8q	86	84	87
2hyw	1	91	90	2i3p	63	64	62	2iff	3	4	4
2o3k	92	91	91	2oka	83	81	84	2omt	90	89	89
2otb	94	93	93	2p0g	86	3	87	2pby	91	6	89
2qu5	80	79	79	2sar	91	39	92	6ebx	88	14	87
6rhn	91	91	90	**3nng**	**24**	**17**	**1**	3npg	7	3	2
3nr6	2	3	4	3o8s	84	2	1	3on5	71	67	68
3q6o	88	40	20	3tx8	15	9	4	3zyt	81	2	2
4e2t	86	82	86	4fqd	77	9	7	1cgn	86	88	90
1cgo	90	90	89	1e8a	87	86	90	2f8m	92	90	93
5ww0	90	86	87	**1vkf**	**2**	**0**	**90**	1vki	95	90	95
1vl2	90	89	91	1vl7	90	90	90	1vlc	90	90	86
2wu6	87	87	87	2x7h	89	89	89	3e49	89	6	90
3gp0	90	89	88	3h9e	89	88	90	3h9r	76	79	77
3khu	88	88	88	3l23	90	90	89	3llx	85	86	85
3m7a	87	88	87	3mbj	85	88	81	3mcq	82	84	83
3mdo	91	88	90	3mz2	1	2	1	3nyy	84	73	78
3obi	1	2	87	3oz2	78	79	78	3p94	89	2	90
3ufi	69	45	48	3us5	93	94	94	4e2e	87	89	87
4ef2	88	47	88	4ezg	89	89	89	4fvs	1	2	1
4gbs	79	31	76	4gcm	88	88	88	4ler	93	92	93
4mru	90	88	90	4ogz	85	6	88	4ouq	87	86	87
4q1v	90	90	90	4q34	90	91	91	4q53	73	73	74
4q6k	93	9	93	4q9a	2	4	75	4qjr	77	74	75
4qni	74	77	74	4r0k	91	77	91	4rvo	1	1	24
4rwv	81	82	85	4yod	79	83	83	**1s45**	**64**	**37**	**75**
1s47	19	10	3	2b1d	10	6	5	2htt	77	---	74
3ce5	58	56	31	3eil	85	46	11	3gom	91	84	5
3goo	91	94	93	3n4o	61	68	50	3tok	45	45	45
4gsg	41	---	44	4l24	70	87	84	4ltl	73	75	77
4ms5	45	5	1	4wo3	26	24	3	4xqz	92	93	3
4zym	1	6	2	5cv2	1	23	3	5i4s	66	---	52
5ihd	93	92	3	5j0e	81	38	54	5ju4	90	100	2

5lj4	84	---	93	5mvt	98	95	86	5nt5	98	88	71
5t4w	95	95	96	5tgp	99	65	1	5ua3	42	26	9
6f3c	59	70	60	6h5r	47	45	44	6tzq	84	84	86
**1iha**	**82**	**81**	**2**	1lc4	86	74	69	1mwl	81	76	78
1q96	2	1	2	1z7f	99	96	99	2a0p	99	99	99
2fd0	89	84	85	2pn4	77	28	91	3d2v	88	44	89
3fs0	92	---	78	3owi	82	73	76	3oxm	89	76	75
3s49	86	1	1	3td1	85	81	81	4enc	90	82	80
4jab	92	81	85	5fj0	1	9	11	5kvj	66	27	57
5l4o	56	62	61	5nz6	62	59	56	5ux3	1	1	1
5uz6	92	19	3	5zeg	19	1	3	6az4	78	74	77
6cab	36	39	45								

SIR22 directives for refining PHASER or MOLREP data using SYNERGY+CAB segments

If the users prefer to use PHASER or MOLREP as an MR program, they will need to provide a few additional directives to process their data through the segments SYNERGY+CAB. As an example, we will use the 1aki structure and MOLREP as the MR program:

%cab buccaneer

%structure 1aki

%job ORTHORHOMBIC FORM OF HEN EGG-WHITE LYSOZYME AT 1.5 Å RESOLUTION

%data

mtz 1aki.mtz

label H K L F SIGF

sequence 1aki.seq

%refine

model 1aki_MolRep.pdb

mtz 1aki_MolRep.mtz

label H K L F SIGF PHIC_ALL FOM

%end
